# Identifying Drug Targets in Pancreatic Ductal Adenocarcinoma Through Machine Learning, Analyzing Biomolecular Networks, and Structural Modeling

**DOI:** 10.3389/fphar.2020.00534

**Published:** 2020-04-30

**Authors:** Wenying Yan, Xingyi Liu, Yibo Wang, Shuqing Han, Fan Wang, Xin Liu, Fei Xiao, Guang Hu

**Affiliations:** ^1^Center for Systems Biology, Department of Bioinformatics, School of Biology and Basic Medical Sciences, Soochow University, Suzhou, China; ^2^State Key Laboratory of Radiation Medicine and Protection, Soochow University, Suzhou, China

**Keywords:** pancreatic ductal adenocarcinoma, drug targets, support vector machine–recursive feature elimination, protein-protein interactions, structural dynamics, integrins

## Abstract

Pancreatic ductal adenocarcinoma (PDAC) is one of the leading causes of cancer-related death and has an extremely poor prognosis. Thus, identifying new disease-associated genes and targets for PDAC diagnosis and therapy is urgently needed. This requires investigations into the underlying molecular mechanisms of PDAC at both the systems and molecular levels. Herein, we developed a computational method of predicting cancer genes and anticancer drug targets that combined three independent expression microarray datasets of PDAC patients and protein-protein interaction data. First, Support Vector Machine–Recursive Feature Elimination was applied to the gene expression data to rank the differentially expressed genes (DEGs) between PDAC patients and controls. Then, protein-protein interaction networks were constructed based on the DEGs, and a new score comprising gene expression and network topological information was proposed to identify cancer genes. Finally, these genes were validated by “druggability” prediction, survival and common network analysis, and functional enrichment analysis. Furthermore, two integrins were screened to investigate their structures and dynamics as potential drug targets for PDAC. Collectively, 17 disease genes and some stroma-related pathways including extracellular matrix-receptor interactions were predicted to be potential drug targets and important pathways for treating PDAC. The protein-drug interactions and hinge sites predication of ITGAV and ITGA2 suggest potential drug binding residues in the Thigh domain. These findings provide new possibilities for targeted therapeutic interventions in PDAC, which may have further applications in other cancer types.

## Introduction

Pancreatic ductal adenocarcinoma (PDAC) is one of the most malignant solid tumors (Bailey et al., 2016). PDAC is difficult to treat due to the stage of diagnosis, severe cachexia and poor metabolic status, the resistance of cancer stem cells (CSCs) to current drugs, and the marked desmoplastic response that facilitates growth and invasion, provides a physical barrier to therapeutic drugs, and prevents immunosurveillance (Al Haddad and Adrian, 2014). PDAC is also a drug-resistant disease, and the response of pancreatic cancer to most chemotherapy drugs is poor. Until now, most of research effort in PDAC has been directed at identifying the important disease-driving genes and pathways (Waddell et al., 2015). These studies have shown that *KRAS*, *CDKN2A*, *TP53*, and *SMAD4* are the four most common driver genes in PDAC (Carr and Fernandez-Zapico, 2019). With the development of multi-omics data, a series of new regulators that are strongly correlated with survival have been proposed to be PDAC biomarkers (Rajamani and Bhasin, 2016; Mishra et al., 2019), including genes (e.g., *IRS1*, *DLL1*, *HMGA2*, *ACTN1*, *SKI*, *B3GNT3*, *DMBT1*, and *DEPDC1B*) and lncRNAs (e.g., PVT1 and GATA6-AS). The integrated transcriptomic analysis of five PDAC datasets identified four-hub gene modules, which were used to build a diagnostic risk model for the diagnosis and prognosis of PDAC (Zhou et al., 2019). Integrated genomic analysis of 456 PDAC cases identified 32 recurrently mutated genes that aggregate into 10 pathways: KRAS, TGF-β, WNT, NOTCH, ROBO/SLIT signaling, G1/S transition, SWI-SNF, chromatin modification, DNA repair, and RNA processing (Bailey et al., 2016). Previous treatments for pancreatic cancer have focused on targeting some of these PDAC-associated pathways, including TGFβ (Craven et al., 2016), PI3K (Conway et al., 2019), Src (Parkin et al., 2019), and RAF→MEK→ERK (Kinsey et al., 2019) and NFAT1-MDM2-MDMX (Qin et al., 2017) signaling, as well as cell-cell communication within the tumor microenvironment (Shi et al., 2019). The discovery of novel drug targets provides extremely valuable resource towards the discovery of drugs. Although the human genome comprises approximately 30,000 genes, proteins encoded by fewer than 400 are used as drug targets in disease treatments. A range of therapeutic targets in PDAC have been proposed, including suppressing the abovementioned genes and pathways (Tang and Chen, 2014). However, the current drug targets for PDAC will not be 100% effective due to the heterogeneous nature of the disease. To tackle this challenge, a complete understanding of the molecular mechanism of PDAC is urgently needed.

Improving PDAC therapy will require a greater knowledge of the disease at both the systems and molecular levels. At the systems level, protein-protein interaction (PPI) networks provide a global picture of cellular function and biological processes (BPs); thus, the network approach is used to understand the molecular mechanisms of disease, particularly in cancer (Conte et al., 2019; Sonawane et al., 2019). Some proteins act as hub proteins that are highly connected to others, thus cancer drug targets can be predicted by hubs in PPI networks (Li et al., 2018; Lu et al., 2018; Zhu et al., 2019). However, there are some conflicting results that suggest disease genes or drug targets have no significant degree of prominence (Mitsopoulos et al., 2015), but higher betweenness, centrality, smaller average shortest path length, and smaller clustering coefficient (Zhao and Liu, 2019). Recent advances in systems biology have led to a plethora of new network-based methods and parameters for predicting essential genes (Li et al., 2019), disease genes, and drug targets (Csermely et al., 2013; Vinayagam et al., 2016; Zhang et al., 2017; Fotis et al., 2018; Liu et al., 2018). Additionally, the structural annotation of PPI networks that has highlighted key residues has enriched the fields of both systems biology and rational drug design (Kar et al., 2009; Winter et al., 2012). The prediction of binding sites, allosteric sites, and genetic variations based on systems-level data is critical for suggesting therapeutic approaches to complex diseases and personalized medicine (Duran-Frigola et al., 2013; Yan et al., 2018). Combined with PPI network analysis, molecular docking studies of target genes can further help to find drug molecules and protein-drug interactions for lung adenocarcinoma (Selvaraj et al., 2018).

Together with advances in “-omics” data, including gene expression and PPI data, machine learning (ML), and artificial intelligence (AI) techniques are powerful tools that can assess gene and protein “druggability” from such massive and noisy datasets (Kandoi et al., 2015; Zhavoronkov, 2018). As the most used ML method, support vector machine (SVM) has been used for cancer genomic classification or subtyping, which may be useful for obtaining a better understanding of cancer driver genes and discovering new biomarkers and drug targets (Huang et al., 2018). ML-based methods have been applied to study PDAC for different purposes. By applying ML algorithms to proteomics and other molecular data from The Cancer Genome Atlas (TCGA), two subtypes of pancreatic cancer can be classified (Sinkala et al., 2020). A meta-analysis of PDAC microarray data could help predict biomarkers that can be used to build AI-based computational predictors for classifying PDAC and normal samples (Bhasin et al., 2016), as well as predicting sample status (Almeida et al., 2020). To predict and validate novel drug targets for cancer, including PDAC, a ML-based classifier that integrates a variety of genomic and systems datasets was built to prioritize drug targets (Jeon et al., 2014).

**Figure 1:**
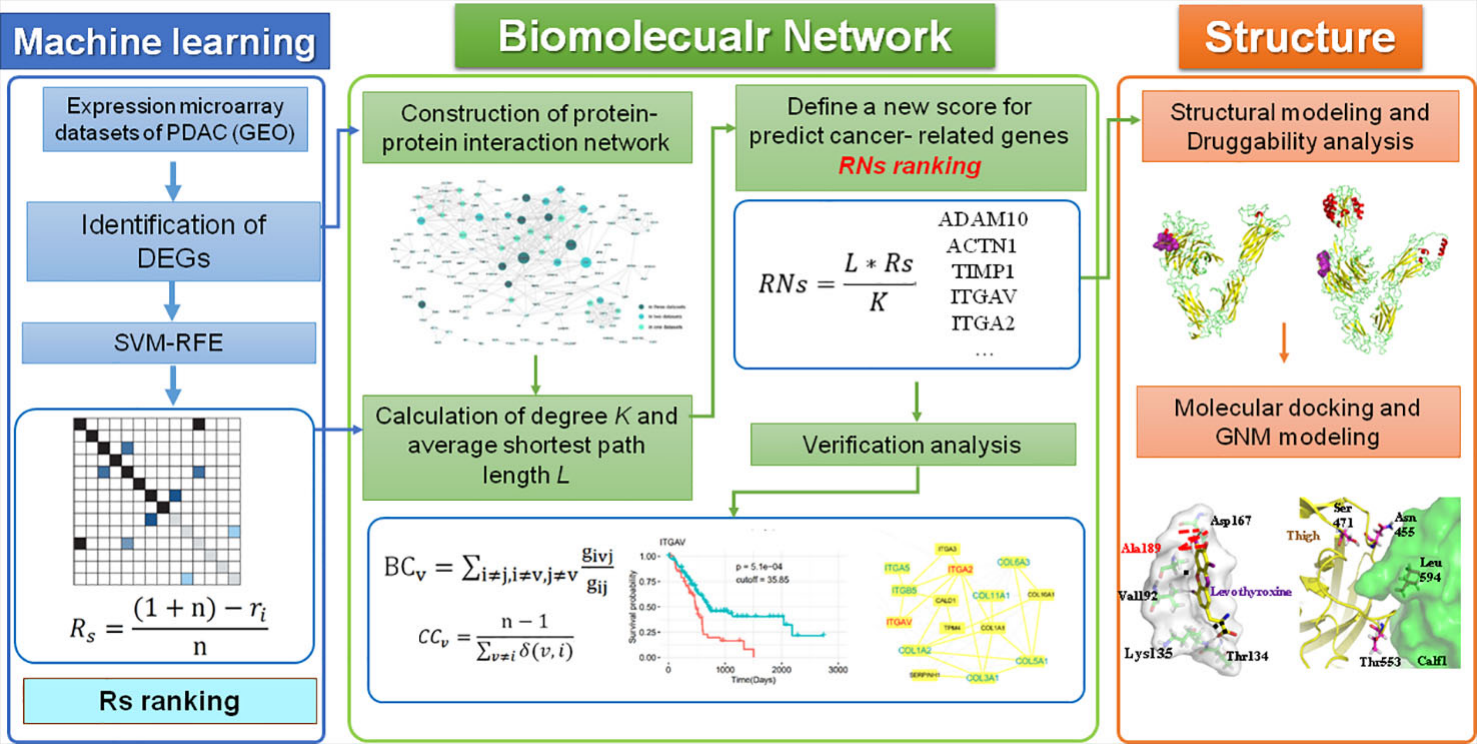
The computational pipeline proposed in this work included three steps. Overall, a machine learning method was used to identify DEGs in PDAC, which were then combined with two parameters of the PPI network to define a new score that predicted disease genes and drug targets in PDAC. All potential targets were then further verified by other bioinformatics analyses and investigated by a “druggability” analysis of structural and dynamic properties.

In this study, we developed a computational framework that integrates various types of high-throughput data, including transcriptomics, interactomics, and structural data, for the genome-wide identification of therapeutic targets in PDAC. A novel centrality metric, referred to as SVM-REF and Network topological score (RNs), was proposed for the identification of disease genes and drug targets. This method incorporates gene expression and network topology information from ML and PPI analyses. Moreover, the predicted genes were validated by “druggability” prediction, survival, and comparative network analyses, as well as functional enrichment analysis. Finally, the structural and dynamic properties of two integrins (ITGAV and ITGA2) as drug targets were investigated. The workflow of these methods is shown in **Figure 1**.

## Materials and Methods

### Identification of DEGs

In this study, three independent PDAC expression microarray datasets with 184 pancreas samples (95 cancer and 89 nonmalignant samples) were used. The datasets were obtained from the National Center for Biotechnology Information (NCBI) Gene Expression Omnibus (GEO, https://www.ncbi.nlm.nih.gov/geo/). Details of each dataset are listed in **Table 1**. The GSE15471 dataset included 36 PDAC samples and matching normal pancreas samples from pancreatic cancer patients in Romania (Badea et al., 2008). There were also matched samples in the GSE28735 dataset, which contains gene expression profiles of 45 matched pairs of pancreatic tumor and adjacent non-tumor tissues from PDCA patients in Germany (Zhang et al., 2012; Zhang et al., 2013). The GSE71989 dataset contained expression profiles of eight normal pancreas and 14 PDAC tissues (Jiang et al., 2016). The normalized data were downloaded from GEO and then analyzed to identify DEGs using t-tests, with p-values adjusted by the Benjamini-Hochberg method. Only genes with adjusted p-values < 0.01 and |FC| > 1.5 were chosen as DEGs.

**Table 1:** Information on the included GEO datasets.

Accessions	Platforms	Samples (tumor vs. non-tumor tissues)	References
**GSE15471**	Affymetrix Human Genome U133 Plus 2.0 Array	36 vs. 36	(Badea et al., 2008)
**GSE28735**	Affymetrix Human Gene 1.0 ST Array	45 vs. 45	(Zhang et al., 2012; Zhang et al., 2013)
**GSE71989**	Affymetrix Human Genome U133 Plus 2.0 Array	14 vs. 8	(Jiang et al., 2016)

### Gene Prioritization Pipeline

Disease genes and drug targets usually have large degree in PPI networks, but there is no single network parameter that can accurately predict them (Li et al., 2016). Protein targets do not exert their function in isolation; rather they are affected by interactions within their PPI network, which are governed by protein localization and environment. In the same way, topological information from PPI networks alone is not enough to identify disease genes and drug targets without biological information. To overcome these limitations, we developed a new three-step pipeline to identify cancer-related genes that may be candidate drug targets in PDAC. The pipeline integrated information from gene expression data and local and global topological characteristics of genes in PPI networks.

**Step 1**: For each gene expression dataset, we employed SVM methods based on a Recursive Feature Elimination (SVM-RFE) algorithm (Guyon et al., 2002), which is an embedded method to specifically deal with gene selection for cancer classification (Bolón-Canedo et al., 2014), rank DEGs, and select the most relevant features (Jeon et al., 2014). SVM-RFE can remove redundant features (genes) to generalize performance, implement backward feature elimination, search an optimal subset of genes, and provide a ranking for each gene. We ranked genes by SVM-RFE score (*R_s_*), according the following formula:

$$R_{S}=\frac{\left(1+\text{n}\right)-r_{i}}{\text{n}},$$

where *n* is the number of DEGs and *r_i_* is the rank of gene *i*.

**Step 2**: A PPI network of DEGs was constructed with the STRING database (von Mering et al., 2003; Szklarczyk et al., 2017) using scores > 0.9. The topological parameters degree and shortest path length for each gene in the PPI network were calculated. The degree (*K*) of a node in the PPI network is the number of links attached to that node, which is one of the measures of centrality of a node in the network. The average path length (*L*) of node *v* in the network is the average length of the shortest paths between *v* and all other nodes and was defined as:

$$L_{v}=\frac{\Sigma_{v\neq i}^{n}\delta \left(v,\;i\right)}{n-1},$$

where *δ*(*v*,*i*) is the length of the shortest path between nodes *v* and *I*, and *n* is the node number in the network.

**Step 3**: Finally, we incorporated Network topological properties into *R_s_* and defined a new score (*RNs*) for each gene as:

$$RNs=\frac{L*R_{s}}{K}.$$

Accordingly, this new *RNs* score (SVM-RFE and Network topological score) considers the cancer status of each gene by including information about gene expression and two levels of topological features in PPI networks, namely, degree *K* indicates the importance of the node, while the shortest path length *L* shows the effects from other nodes. The code for gene prioritization is freely available on GitHub for download at: https://github.com/CSB-SUDA/RNs.

### PPI Network Analysis

Once the PPI network was constructed, two other analyses were performed. The first analysis was the calculation of two commonly used centrality parameters: betweenness and closeness centrality. The betweenness centrality (BC) (Freeman, 1977) of node *v* was defined as:

$${\text{BC}}_{\text{v}}=\Sigma_{\text{i}\neq \text{j},\text{i}\neq \text{v},\text{j}\neq \text{v}}\frac{g_{ivj}}{g_{ij}},$$

where *g_ivj_* is the number of the shortest paths from *i* to *j* that pass through node *v*, and *g_ij_* is the number of shortest paths from *i* to *j*.

The closeness (CC) of node *v* is the reciprocal of the average shortest path length, which was calculated as:

$$CC_{v}=\frac{\text{n}-1}{\Sigma_{v\neq i}\delta (v,i)}.$$

Proteins are often incorporated into modules that can be shared between several different cellular activities. The second analysis was module detection of PPIs by integrating a Gaussian network (GN) algorithm (Newman and Girvan, 2004) and functional semantic similarity (Wang et al., 2007). In general, this involved using the GN algorithm to detect the module of PPI networks, and then applying functional semantic similarity to filter links. Thus, the genes in the detected modules not only had topological similarity, but also functional similarity.

### Survival Analysis

To evaluate the prognostic value of candidate genes, a survival analysis was performed using data from the human protein atlas (Uhlen et al., 2017), which contains gene expression data and clinical information of 176 pancreatic cancer patients. P-values < 0.01 were considered significantly correlated with overall survival.

### Functional Enrichment Analysis

Functional enrichment analysis, including cellular component (CC), molecular function (MF), and BP, from the Kyoto Encyclopedia of Genes and Genomes (KEGG) pathways of genes was performed using the R package cluster Profiler (Yu et al., 2012). Terms with adjusted p-value < 0.05 were considered significant.

### Structural Modeling and “Druggability” Analysis

The protein structures of potential drug targets were retrieved from the Protein Data Bank (PDB) if they were available. The Swiss model (Waterhouse et al., 2018) and I-TASSER (Roy et al., 2010) were used for the structural modeling of genes if protein structures were unavailable. We choose the Swiss model when the sequence similarity between searched models was >30%; otherwise, we used I-TASSER, which predicts protein structure using modeling by iterative threading assembly. Based on model structures, Fpocket (Le Guilloux et al., 2009) was used to detect druggable pockets and calculate “druggability” scores, which were based on several physicochemical descriptors on a genomic scale. The pocket with the highest score in the entire PDB was defined as the reference druggable score. The score of each pocket was classified as: 0.0–0.5: non-druggable; 0.5–0.7: druggable; and 0.7–1.0: highly druggable.

### Molecular Docking and GNM Modeling

To study the interactions and binding mode of small molecules with the potential drug targets, molecular docking was performed using AutoDock 4.2 (Khodade et al., 2007). The target, drug, and related disease information were collected from the Drug Bank database (Version 5.0) (Wishart et al., 2018) and the Therapeutic Target Database 2020 (Wang et al., 2020). A normal mode analysis of the GN model (GNM) was performed to investigate collective dynamics *via* the DynOmics online tool (Danne et al., 2017). The default cutoff distance of 7.3 Å between GNM model nodes was used.

## Results and Discussion

### Identification of Disease Genes and Drug Targets in PDAC

**Figure 2:**
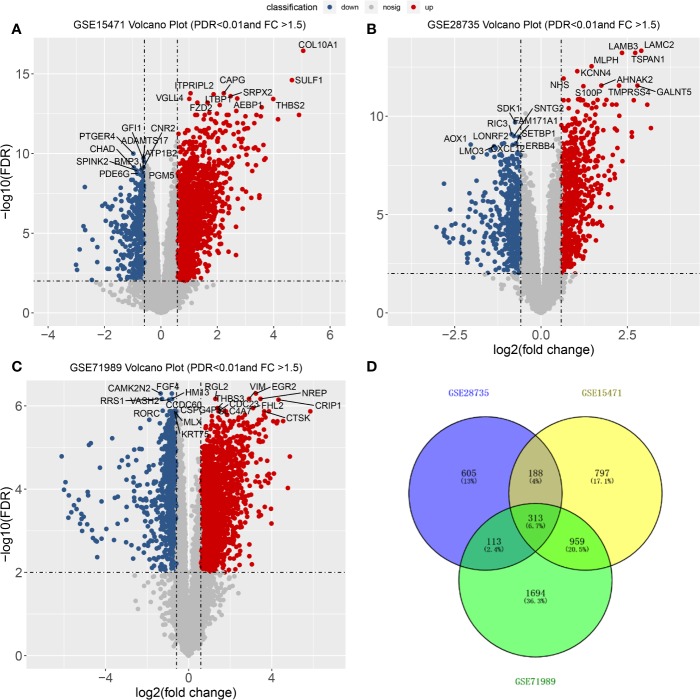
Differentially expressed genes (DEGs) between PDACs and normal tissues. **(A–C)** Volcano plot of −log10 (FDR) vs. log2 (fold change) of DEGs in the three datasets. **(D)** Venn diagram with the number of overlapping DEGs from the different datasets.

From the three datasets GSE28735, GSE71989, and GSE15471, we identified 3,079, 1,225, and 2,257 DEGs between PDAC and adjacent tissues, respectively. The top 10 genes with the smallest p-values are marked in **Figure 2**. In GSE28735, 1,724 genes showed increased expression in PDAC tissues, while 1,355 genes showed decreased expression (**Figure 2A**). In GSE71989, 766 genes were upregulated and 459 genes were downregulated in PDAC tissues compared with normal tissues (**Figure 2B**). In GSE15471, 1713 genes were overexpressed, while 544 genes showed decreased expression in tumor tissues (**Figure 2C**). Together, there were 313 common DEGs between PDAC and adjacent tissues in all three datasets (**Figure 2D**).

Additionally, we evaluated gene expression as an input feature for ML and selected the most relevant genes for PDAC using SVM-RFE (Almeida et al., 2020), which provided a ranking for the genes. Then, each DEG was assigned an *R_s_* value (see *Materials and Methods*), which was used to further rank all genes. As an illustration, the top 100 *R_s_* values of the DEGs in each dataset are listed in **Table S1**. This shows that there is little overlap of results between the different datasets. This means that calculating *R_s_* based on SVM-RFE can provide information for classification, but not enough for ranking.

**Figure 3:**
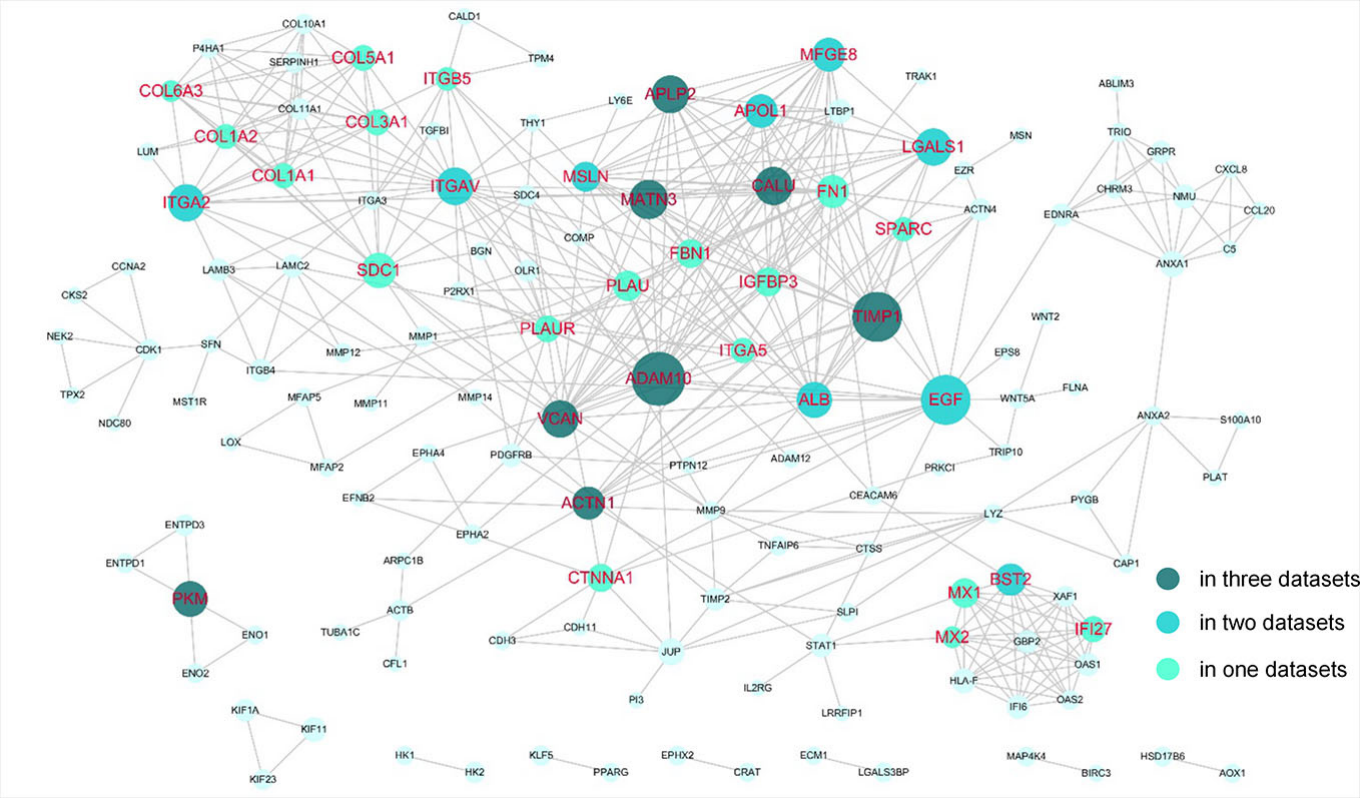
Potential drug targets in the PPI network. The genes that were predicted by our pipeline are marked with red labels. The node size denotes the average RNs of the gene in two or three datasets.

The DEGs were next mapped to the STRING database, which yielded a PPI network with 144 genes and 440 links (**Figure 3**). Then, degree and shortest path length of each gene in the network were calculated. Finally, we ranked the genes according to our designed score RNs, which integrated these two topological parameters and was based on gene expression profile. The top 20 genes predicted based on at least two datasets were considered potential drug targets. As shown in **Table 2** and **Table S2**, eight genes (*ADAM10, TIMP1, MATN3, PKM, APLP2, ACTN1, CALU*, and *VCAN*) were identified in all three datasets, and nine genes (*LGALS1*, *ITGA2*, *BST2*, *MFGE8*, *ITGAV*, *EGF*, *APOL1*, *ALB*, and *MSLN*) were identified in two of three datasets. We propose that genes predicted by at least two datasets could serve as disease genes and/or drug targets. Taken together, 17 genes predicted by RNs score are listed in **Table 3**, and most have been previously reported to be PDAC-associated genes. There are only four that have not been previously associated with PDAC. This suggests that our metric RNs is useful for identifying novel disease genes and drug targets.

**Table 2:** Identified potential drug targets for PDAC.

**In three datasets**	*ADAM10*, *TIMP1*, *MATN3*, *PKM*, *APLP2*, *ACTN1*, *CALU*, *VCAN*
**In two of three datasets**	*LGALS1*, *ITGA2*, *BST2*, *MFGE8*, *ITGAV*, *EGF*, *APOL1*, *ALB*, *MSLN*
**In only one dataset**	*COL5A1*, *CTNNA1*, *MX1*, *COL1A2*, *COL6A3*, *SPARC*, *IFI27*, *SDC1*, *FN1*, *PLAU*, *PLAUR*, *IGFBP3*, *FBN1*, *COL1A1*, *COL3A1*, *ITGB5*, *ITGA5*, *MX2*

**Table 3:** List of prioritized protein targets with their drug target information and “druggability” features.

Gene	RNs	Drug targets^*^	Drug(s)^#^	Disease(s)^#^	PDB	DS
**ADAM10**	5.34	Yes	XL784	Solid tumor/cancer, Breast cancer	6BE6	0.694
**TIMP1**	4.79	No	NA	NA	1LQN	0.839
**EGF**	4.77	Yes	Sucralfate, Tesevatinib, Alpha-Aminobutyric Acid, Cholecystokinin	Oral mucositis, Vulnerary	template: 5GJE	0.968
**MATN3**	3.31	No	NA	NA	template: 6BXJ	0.545
**CALU**	3.17	No	NA	NA	template: 2Q4U	0.677
**APLP2**	3.12	Yes	Zinc, Zinc acetate, Zinc chloride	NA	5TPT	0.912
**ITGAV**	3.08	Yes	Abituzumab, Levothyroxine	Colorectal cancer, Solid tumour/cancer	3IJE	0.663
**VCAN**	3.05	Yes	Hyaluronic acid	NA	template: 4CSY	NA
**LGALS1**	3.03	Yes	Thiodigalactoside, 1,4-Dithiothreitol, Mercaptoethanol, Artenimol	NA	3W59	NA
**ITGA2**	3.027	No	NA	NA	Templates: 3K71, 4NEH, 3K6S	0.672
**ALB**	2.85	Yes	Gadobenate Dimeglumine, Glycyrrhizic acid, Patent Blue, (365 drugs)	Hemophilia, Schizophrenia	4BKE	1.000
**PKM**	2.82	Yes	Pyruvic acid, L-Phospholactate, 2-Phosphoglycolic Acid, et al.	Pain, Renal cell carcinoma;	6GG5	0.996
**MFGE8**	2.54815	No	NA	NA	template: 4DEQ	NA
**APOL1**	2.52	Yes	Zinc, Zinc acetate, Zinc chloride	NA	template: 5J2L	0.503
**ACTN1**	2.45	Yes	Copper, Human calcitonin	NA	template: 4D1E	0.673
**BST2**	2.31	No	NA	NA	3MQB	0.821
**MSLN**	2.0	Yes	Amatuximab	Ovarian/Pancreatic cancer	4F3F	0.727

*”YES” means drug target, and “NO” means non-drug target; ^#^”NA” means no drug and disease information, or no druggable pockets.

It is also useful to compare our results predicted by RNs with other common network parameters. The genes predicted by calculating betweenness and closeness centrality are also listed in **Table S2**. Among our 20 predicted potential drug targets, six and nine were also found by betweenness and closeness centrality, respectively. Notably, *ADAM10*, *ACTN1*, and *TIMP1* were in all three lists, which suggested they had important roles in PDAC. Moreover, two other genes (*ITGAV* and *ITGA2*) were in the top 20 of two datasets, which suggested they should be investigated. Overall, compared with the top 20 genes predicted by these two common network parameters, our RNs parameter identified more extracellular matrix (ECM) proteins, including integrins and collagens. The other interesting finding was that four common genes (*ALB*, *EGF*, *ITGA2*, and *VCAN*) were identified by isolating the nodes with large degrees (hubs) in PPI network construction based on other PDAC GSE datasets (Lu et al., 2018).

**Figure 4:**
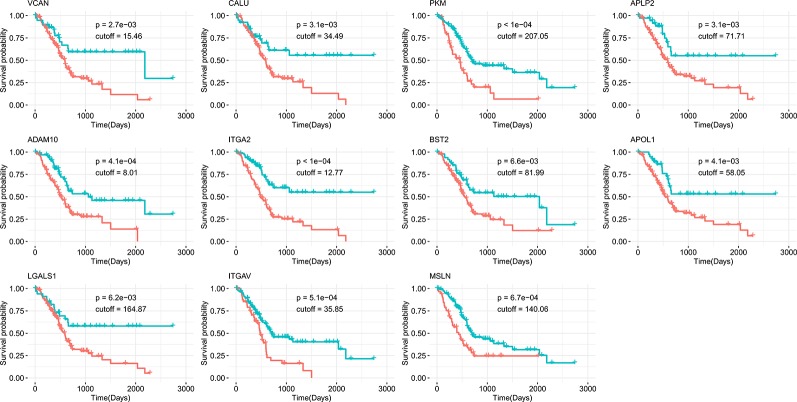
Kaplan-Meier survival curves of overall survival from the human protein atlas datasets for potential drug targets divided by high (red) or low (green) expression level.

Survival analysis was also performed to evaluate whether the expression of our 17 identified candidates was related to the prognosis of PDAC. Using Kaplan-Meier analysis with the log-rank test for 176 pancreatic cancer patients from the human protein atlas (Uhlen et al., 2017), we found that higher expression levels of 11 genes were significantly correlated with decreased overall survival (p < 0.01, **Figure 4**). For the eight genes identified in all three datasets, five (*ADAM10*, *PKM*, *APLP2*, *CALU*, and *VCAN*) were associated with poor prognosis when highly expressed. The other six highly expressed genes (*LGALS1*, *ITGA2*, *BST2*, *ITGAV*, *APOL1*, and *MSLN*) associated with poor prognosis that were identified in two of three datasets are shown in **Table 2**. Accordingly, the survival analysis showed significant prognostic values for most of the predicted genes.

### Characterization of Predicted Drug Targets for PDAC

**Table 3** shows the genes predicted above shortlisted based on our RNs criteria. After searching the drug bank, these 17 predicted genes were classified into two types: 11 genes were drug targets, while six were non-drug targets. We also annotated drug targets in the drug bank by their related drugs and diseases. It should be noted that *MSLN* was the only proven drug target for PDAC, and there are many drugs that inhibit *ALB*. Thus, we concluded that these two genes had been studied widely and would not give us more insight regarding discovering new targets. Considering the potential of other predicted genes as drug targets for PDAC, we performed functional and “druggability” annotations for all. Among the 15 genes, 11 (*ADAM10*, *TIMP1*, *EGF*, *APLP2*, *ITGAV*, *VCAN*, *ITGA2*, *PKM*, *APOL1*, *ACTN1*, and *BST2*) have been reported to be contributing factors in PDAC invasion, growth, or metastasis, which indicated that our pipeline had good performance for finding potential drug targets for PDAC.

The protease *ADAM10* was predicted as the highest ranked gene, and it has been reported that *ADAM10* influences the progression and metastasis of cancer cells, as it promotes PDAC cell migration and invasion (Gaida et al., 2010). Inhibiting *ADAM10* could be a novel approach for natural killer (NK) cell-based immunotherapy (Pham et al., 2017). Tissue inhibitor of metalloproteinases-1 (*TIMP-1*) correlated with tumor progression, and elevated levels of *TIMP-1* in tumor tissue and peripheral blood were associated with poor clinical outcomes in numerous malignancies, including PDAC (Prokopchuk et al., 2018). The third gene was epidermal growth factor (*EGF*), which was a common disease gene for many cancers, and EGF mutations were associated with PDAC (Grapa et al., 2019). Amyloid precursor-like protein 2 (*APLP2*) affects the actin cytoskeleton and also increases PDAC growth and metastasis (Pandey et al., 2015). *ITGAV* (Villani et al., 2019), *VCAN* (Skandalis et al., 2006), and *ITGA2* (Nones et al., 2014) are matrix proteins that have been shown to contribute to pancreatic cancer cell migration, invasion, and metastasis. *PKM2* is one of the isoforms of pyruvate kinase muscle isozyme (*PKM*) and promotes the invasion and metastasis of PDAC through the phosphorylation and stabilization of PAK2 (Cheng et al., 2018). The final three genes, *APOL1* (Liu et al., 2017), *ACTN1* (Rajamani and Bhasin, 2016), and *BST2* (Grutzmann et al., 2005) have previously been reported to be effective biomarkers for PDAC.

Although 11 genes were already known drug targets, “druggability” annotations based on protein structures can improve our knowledge and understanding of the mechanisms of proteins as drug targets. The “druggability” of proteins is a measure of their ability to bind drug-like molecules based on molecular shapes. For the “druggability” of all 17 genes, we first obtained their structural modes by retrieved data from the PDB database or homology modeling. The PDB codes of proteins or their templates are listed in **Table 3**. Then, Fpocket was used to compute all possible pockets and their corresponding “druggability score” (DS). The “druggability” of the protein was defined as the DS of the highest scoring pocket. As expected, most of the predicted proteins were druggable (DS ≥ 0.5), except VCAN, IGALS1, and MFGE8. ALB had the largest DS (1.00), which can partially explain why so many ALB inhibitors exist. Among the six non-drug targets, TIMP1, ITGA2, and BST2 were predicted as highly druggable (DS ≥ 0.5), which meant that these three genes had the structural abilities to be drug targets. In particular, the non-drug target ITGA2 had a larger DS than ITGAV, suggesting that a more detailed structural comparison between these two integrin proteins is needed.

### Identification of Functional Modules and Pathways

**Figure 5:**
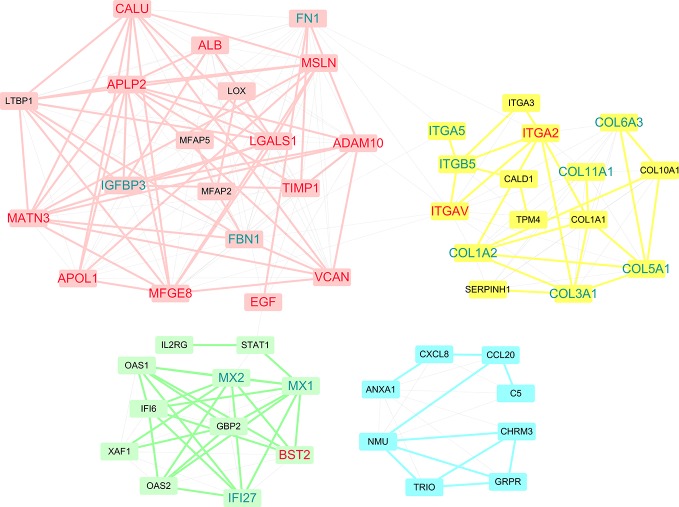
Four modules were discovered within PPI networks. Genes that were predicted in at least two datasets are marked red, while genes that were predicted in only one dataset are marked blue.

Within PPI networks, cancer targets interact with different modules to perform biological functions. A module within a network is defined a set of nodes that are densely connected within subsets of the network but may not all directly interact with each other. To get further insight into the topological and biological functions of potential targets, we performed module detection in the PPI network using a GN algorithm and functional semantic similarity. As shown in **Figure 5**, we identified four modules (the pink, yellow, green, and blue nodes) and labeled the genes that were predicted in at least two datasets (red) or in only one dataset (blue). Except *PKM* and *ACTN1*, 15 of the 17 predicted genes were detected by the modular analysis and are included in these four modules. The top module (pink) was formed of 19 genes, including the most of our predicted genes (12/17, *ADAM10*, *CALU*, *ALB*, *APLP2*, *MSLN*, *LGALS1*, *TIMP1*, *MATN3*, *VCAN*, *EGF*, *MFGE8*, and *APOL1*). Most of these genes have been previously reported as disease genes in PDAC or drug targets in other cancers. Another three predicted genes were included in two other modules, while *ITGAV* and *ITGA2* were detected in the second largest module (yellow). Although there were only two predicted genes, this module deserves more attention, as it primarily contains two types of gene targets: integrins (*ITGA5*, *ITGA3*, *ITGB5*, *ITGA2*, and *ITGAV*) and collagens (*COL6A3*, *COL11A1*, *COL1A1*, *COL10A1*, *COL5A1*, *COL1A2*, and *COL3A1*). Research into integrins and collagens and their interactions may provide more insights into the molecular mechanisms of PDAC.

**Figure 6:**
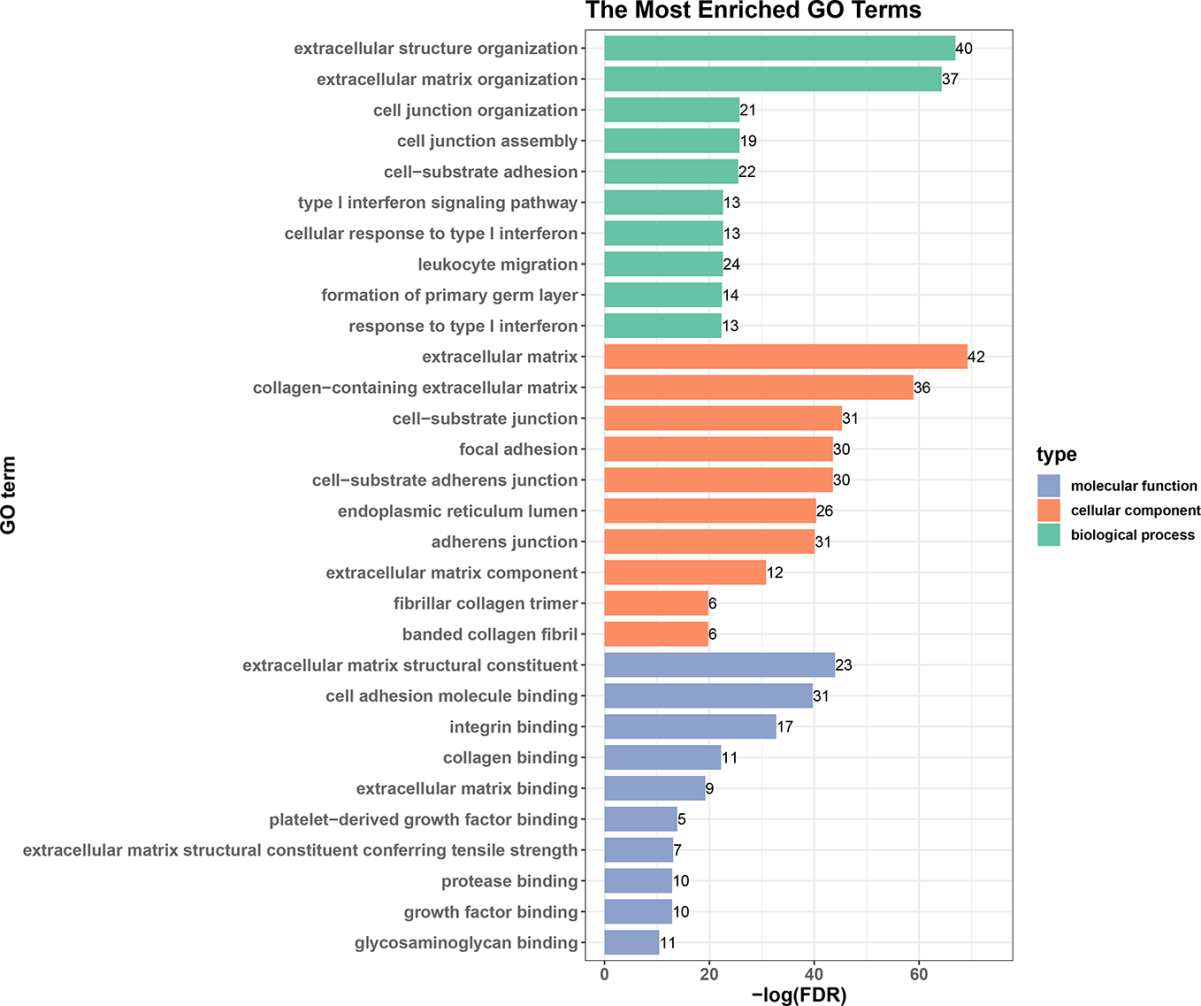
Top 10 enriched GO terms in biological processes, cellular components, and molecular functions.

We next performed an enrichment analysis on genes in the PPI network (**Figure 6** and **Table 4**). The genes were enriched for the GO terms related to extracellular structure and matrix, such as extracellular structure and matrix organization in BP, ECM in CC, and ECM structural constituent and binding in MF. **Table 4** shows the top 10 most significantly enriched KEGG pathways. Most of the pathways are associated with cancer, such as ECM-receptor interaction, focal adhesion, and proteoglycans in cancer. Moreover, integrins were enriched in most of the carcinogenesis-associated pathways, such as focal adhesion, which play essential roles in important BPs, including cell motility, proliferation, and differentiation. Interestingly, several altered molecular pathways were identified, which suggests that genes in the secondary module were involved in these pathways. These modules and pathways not only contained integrins, but also another group of collagens. In particular, two predicted integrins (*ITGAV* and *ITGA2*) were involved in nine out of the top 10 pathways, while the top four pathways (ECM-receptor interaction, focal adhesion, proteoglycans in cancer, and human papillomavirus infection) also contained collagens, especially *COL1A1* and *COL1A2*. Except for these pathways, the list of integrins and collagens was used to define the traditional cancer-related PI3K/AKT pathway. It was previously known that collagen is a major component of the tumor microenvironment that participates in cancer fibrosis, which can influence tumor cell behavior through integrins (Xu et al., 2019). Our results indicated that *ITGAV*, *ITGA2*, and their interactions with *COL1A1* and *COL1A2* may play important roles in PDAC, suggesting they could serve as potential drug targets. For example, the predicted genes and their interactions were highlighted in the ECM-receptor interaction pathway (**Figure S1**). This systems biology evidence of gene cluster- and pathway-based distributions suggested that targeting several key genes together could be a more promising approach.

**Table 4:** Top 10 enriched KEGG pathways (integrins and collagens are marked in bold).

KEGG term	Gene(s)	Count	Adjust p-value
**ECM-receptor interaction**	***COL1A1*, *COL1A2*, *COL6A3***, *COMP*, *FN1*, ***ITGA2***, ***ITGA3***, ***ITGA5*, *ITGAV*, *ITGB4*, *ITGB5***, *LAMB3*, *LAMC2*, *SDC1*, *SDC4*	15	2.62E-11
**Focal adhesion**	*ACTB*, *ACTN4*, *ACTN1*, *BIRC3*, ***COL1A1*, *COL1A2*, *COL6A3***, *COMP*, *EGF*, *FLNA*, *FN1*, ***ITGA2***, ***ITGA3*, *ITGA5*, *ITGAV*, *ITGB4*, *ITGB5***, *LAMB3*, *LAMC2*, *PDGFRB*	20	2.67E-11
**Proteoglycans in cancer**	*ACTB*, ***COL1A1*, *COL1A2***, *FLNA*, *FN1*, ***ITGA2*, *ITGA5*, *ITGAV*, *ITGB5***, *LUM*, *MMP9*, *MSN*, *PLAU*, *PLAUR*, *SDC1*, *SDC4*, *EZR*, *WNT2*, *WNT5A*	19	2.97E-10
**Human papillomavirus infection**	***CCNA2*, *COL1A1*, *COL1A2*, *COL6A3***, *COMP*, *EGF*, *FN1*, *HLA-F*, ***ITGA2*, *ITGA3*, *ITGA5*, *ITGAV*, *ITGB4*, *ITGB5***, *LAMB3*, *LAMC2*, *MX1*, *PDGFRB*, *PKM*, *PRKCI*, *STAT1*, *WNT2*, *WNT5A*	23	4.12E-10
**Regulation of actin cytoskeleton**	*ACTB*, *ACTN4*, *ACTN1*, *CFL1*, *CHRM3*, *EGF*, *FN1*, ***ITGA2*, *ITGA3*, *ITGA5*, *ITGAV*, *ITGB4*, *ITGB5***, *MSN*, *PDGFRB*, *EZR*, *ARPC1B*	17	8.45E-10
**Arrhythmogenic right ventricular cardiomyopathy (ARVC)**	*ACTB*, *CTNNA1*, ***ITGA2*, *ITGA3*, *ITGA5*, *ITGAV*, *ITGB4*, *ITGB5***, *JUP*	9	1.25E-05
**PI3K-Akt signaling pathway**	***COL1A1*, *COL1A2*, *COL6A3***, *COMP*, *EGF*, *EPHA2*, *FN1*, *IL2RG*, ***ITGA2*, *ITGA3*, *ITGA5*, *ITGAV*, *ITGB4*, *ITGB5***, *LAMB3*, *LAMC2*, *PDGFRB*	17	3.87E-05
**Amoebiasis**	*ACTN4*, *ACTN4*, *ACTN1*, ***COL1A1*, *COL1A2*, *COL3A1***, *FN1*, *CXCL8*, *LAMB3*, *LAMC2*	9	1.03E-04
**Hypertrophic cardiomyopathy (HCM)**	*ACTB*, ***ITGA2*, *ITGA3*, *ITGA5*, *ITGAV*, *ITGB4*, *ITGB5***, *TPM4*	8	3.01E-04
**Small cell lung cancer**	*BIRC3*, *CKS2*, *FN1*, ***ITGA2*, *ITGA3*, *ITGAV***, *LAMB3*, *LAMC2*	8	3.01E-04

### ITGAV and ITGA2 as Potential Drug Targets for PDAC

By combining SVM-RFE, PPI network, and survival analysis, 11 out of 17 candidate genes have been predicted as biomarkers in pancreatic cancer patients. Among them, two integrins of ITGAV and ITGA2 were further screened as two potential drug targets according to the following evidences: 1) Both ITGAV and ITGA2 are involved in all PDAC-related pathways include ECM-receptor interaction and focal adhesion pathways, suggesting that ITGAV and ITGA2 may play an important role in PDAC progression; 2) Based on the druggability criteria, ITGAV and ITGA2 have relatively high DS. In addition, ITGAV is already a drug target for other cancer. Due to the structural similarity, ITGA2 can also be considered as a potential drug target; 3) Current experimental data suggest that several other integrins are overexpressed in various cancer types, being involved in tumor progression through tumor cell invasion and metastases. For example, the therapeutic potential of ITGA5 in the PDAC stroma has been proved efficacy (Kuninty et al., 2019). Collectively, our data together with some know results point towards ITGAV and ITGA2 as two potential drug targets for PDAC. Thus, the emerging understanding of their structural properties will guide the development of new strategies for anticancer therapy.

Integrins are transmembrane receptors that are central to the biology of many human pathologies. Classically, integrins are known for mediating cell-ECM and cell-cell interaction, and they have been shown to have an emerging role as local activators of TGF-*β*, influencing cancer, fibrosis, thrombosis, and inflammation (Raab-Westphal et al., 2017). Integrins are composed of *α* and *β* subunits to form a complete signaling molecule. Their ligand binding and some regulatory sites are extracellular and sensitive to pharmacological intervention, as proven by the clinical success of seven drugs that target integrins (Hamidi et al., 2016). Although peptides and small molecules are generally designed to target integrin *αβ* dimers, the individual integrin *α* subunits may also be therapeutic targets. ITGAV always bind with five *β* subunits that form receptors for vitronectin, cytotactin, fibronectin, fibrinogen, and laminin. ITGAV has mostly been investigated for its role in malignant tumor cells and tumor vasculature (Xiong et al., 2001; Xiong et al., 2009). ITGAV recognizes the Arg-Gly-Asp (RGD) sequence in a wide array of ligands at the interface between the α and β subunits (Xiong et al., 2002). ITGA2 forms with *β*_1_ and belongs to the collagen receptor subfamily of integrins (Emsley et al., 2000).

**Figure 7:**
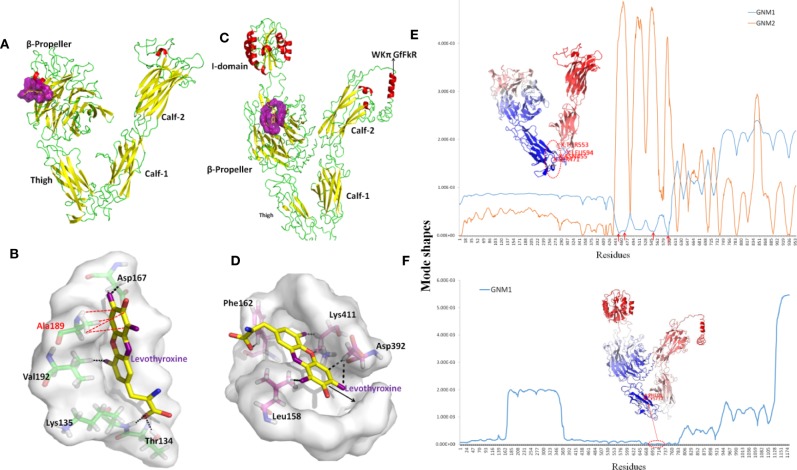
Structures and dynamics of ITGAV and ITGA2. **(A)** The structure of ITGAV including the *β*-propeller, Thigh, Calf-1, and Calf-2 domains, and the most druggable pocket (purple), which is located along the outer side of the *β*-barrel. **(B)** The binding poses by docking Levothyroxine into the most druggable pocket of ITGAV. Levothyroxine and interacting residues are represented as colored sticks. **(C)** The structure of ITGA2 including the *I*-, *β*-propeller, Thigh, Calf-1, and Calf-2 domains, and the most druggable pocket (purple), which is located at the hole of the *β*-barrel; the binding pose with Levothyroxine and this pocket is shown in **(D)**. **(E)** The shapes of first and second GNM modes of ITGAV. The minimum of the shapes indicate the hinge region, which corresponds to the structure in dark blue. Mode 1 predicts Asn455, Ser471, Arg553, and Gly594 within the Thigh domain are hinge sites (red arrows). **(F)** The shape of the first GNM mode of ITGA2, where the region of Phe681 to Ser737 within the Thigh domain was predicted to contain hinge sites (red circle).

The structure of ITGAV was taken from chain A of the *x*-ray structure of complete integrin *αVβ*_3_ (PDB code: 3IJE). It contains a *β*-propeller domain of seven 60-amino-acid repeats, and three other domains including the Thigh, Calf-1, and Calf-2 domains (**Figure 7A**). The PDB repository contains no crystal structure for full-length ITGA2. The highest sequence similarity between ITGA2 and searched models (PDB code: 5ES4) was 28%, so we employed I-TASSER to generate a composite model of ITGA2 based on several templates. A subsequent analysis of the structure of ITGA2 revealed similar domain structures with ITGAV but with the addition of an *I* domain (Emsley et al., 1997) and a *WKπ GfFkR* helix tail, which may suggest more drug-targeting possibilities for ITGA2. Based on the structures of ITGAV and ITGA2, Fpocket was used to detect their druggable pockets. For ITGAV, there were two highly druggable pockets, both located within the *β*-propeller domain. The largest druggable pocket was located on the outer side of the *β*-barrel, consisted of Val192, Lys104, Ala189, Asp132, Val188, Ala189, Asp167, Leu130, Gln187, Glu190, Lys135, Val137, and Gln131, and had a DS of 0.663 (**Figure 7A**). The second largest druggable pocket was located at the hole of the *β*-barrel, consisted of Trp93, Leu111, Gln156, Phe159, Pro110, Ala96, Phe21, Tyr406, Tyr224, and Phe278, and had a DS of 0.599 (**Figure S2A**). For ITGA2, only one highly druggable pocket was found at the *β*-propeller domain and had a DS of 0.92. This pocket consisted of His416, Phe162, His414, Ser159, Phe156, Leu417, Ser161, Val409, Leu396, Lys411, Leu158, Gln157, Leu394, Ala160, Leu417, Asp155, Asp392, Val381, Gly415, and Ser413 (**Figure 7B**).

Despite progress in the development of drugs that target different integrins, there are only two clinical approved drugs in the drug bank for ITGAV (Levothyroxine and Antithymocyte immunoglobulin) (**Table 3**). Thymoglobulin is a polyclonal antibody, while Levothyroxine is currently the only approved small molecule that targets ITGAV. The small ligand Levothyroxine was docked to the two druggable pockets in ITGAV to study the stability of the complex and protein-drug interactions. When docked to the largest druggable pocket, Levothyroxine formed hydrogen bonds with Asp167, Thr134, Lys135, and Val192, and a hydrophobic interaction with Ala189, and the binding free energy was −8.3 kcal/mol (**Figure 7C**). For the other pocket, hydrogen bonds were formed between Levothyroxine and Phe21, Trp93, Ala96, and Pro110 with the binding free energy of −10.08 kcal/mol (**Figure S2B**). We further docked Levothyroxine to ITGA2 at its druggable pocket. The binding free energy of −9.09 kcal/mol suggested a good interaction between ITGA2 and Levothyroxine, with the potential binding sites at Phe162, Lys411, Asp392, and Leu158 (**Figure 7D**).

To determine residues that play a key role in the global dynamics of ITGAV and ITGA2, we performed a GNM analysis. GNM analysis provides information on the mechanisms of collective movements intrinsically accessible to the structure, which usually enable structural changes relevant to function (Bahar et al., 2010). The most discriminative feature in dynamic analysis is hinge prediction, which are expected to be sites for drug development (Sumbul et al., 2015). We predicted hinges sites by the minima of corresponding GNM slow modes. By applying GNM to ITGAV (**Figure 7E**), GNM mode 1 highlights the hinge region located in the Thigh domain, especially at Asn455, Ser471, Arg553, and Gly594, which are located at the interface between the Thigh and Calf-1 domains. We also note that the *β*-propeller domain became the major hinge region in GNM mode 2, while Ile286, Asn287, Asp352, Phe377, Ser389, Thr413, Asp414, Pro421, and Tyr436 have minimal fluctuations. Hinge sites located at the β-propeller domain in GNM mode 2 may correspond to pocket sites, as the first and second largest druggable pockets were within the β-propeller domain. For ITGA2 (**Figure 7F**), both GNM modes 1 and 2 highlighted the same hinge regions within the β-propeller domain and the Thigh domain, with critical positioning of Phe681 to Ser737. Accordingly, our GNM modeling suggested that both the *β*-propeller domain and the Thigh domain play important roles in modulating the collective movements of ITGAV and ITGA2. The *β*-propeller domain has been indicated to be a druggable domain by pocket detection. Here, some hinge sites located within the Thigh domain offer other reasonable starting points for inhibitor design.

## Conclusions

In this study, we developed a computational framework that integrated ML (SVM-RFE), biomolecular networks (PPI network analysis), and structural modeling analysis (homology modeling, molecular docking, and GNM modeling) to help future drug targets for PDAC. The core of the new method was that we defined a new score, termed RNs, based on cancer-related information from gene expression data and topological information obtained from PPI network analysis. Research using three GEO datasets (GSE28735, GSE71989, and GSE15471) yielded 17 genes (*ADAM10*, *TIMP1*, *MATN3*, *PKM*, *APLP2*, *ACTN1*, *CALU*, *VCAN*, *LGALS1*, *ITGA2*, *BST2*, *MFGE8*, *ITGAV*, *EGF*, *APOL1*, *ALB*, and *MSLN*) that were predicted to be potential drug targets. The survival and “druggability” analysis of these genes showed that most of the identified genes had poor survival associations and good DS values, further providing evidence that they can be used as therapeutic targets in PDAC. The important roles of integrins as well as their interactions with collagens were highlighted by combining network modules and KEGG pathway analysis, in term of four pathways, ECM-receptor interaction, focal adhesion, proteoglycans in cancer, and human papillomavirus infection pathways. By focusing on ITGAV and ITGA2, we identified druggable pockets, drug binding sites, and hinge sites that are potential sites for designing small molecules. In summary, this new methodology will provide new avenues for discovering drug targets in PDAC and other cancers.

Of course, our method in this work has some limitations. Firstly, our method only used SVM-REF to the gene expression data to rank the DEGs. With the growth of other omics data, we need to apply our method by including more kinds of data, such as RNA-Seq data for PDAC (Raphael et al., 2017), which will make our method more practical. Secondly, our method just combined the systems level analysis of PPI construction and analysis and the molecular level analysis of “druggability” prediction, and thus, the drug target prediction needs some structural research experience to some extent. To address this, the real integration of structure knowledge into PPI networks is still needed.

## Data Availability Statement

The raw data supporting the conclusions of this article will be made available by the authors, without undue reservation, to any qualified researcher.

## Author Contributions

WY, XYL, and GH analyzed the data and wrote the manuscript. XYL and WY conducted the SVM calculation and network analysis. FW assisted in network analysis. FX and SH conducted the structural modeling and docking. XL assisted in molecular docking. WY, FX, and GH conceived and designed all experiments, and interpreted all results. GH revised the manuscript. All authors contributed to the work.

## Funding

This study was supported by the National Natural Science Foundation of China (31872723), the Project of State Key Laboratory of Radiation Medicine and Protection, Soochow University (No. GZK1201902), and a project funded by the Priority Academic Program Development (PAPD) of Jiangsu Higher Education Institutions

## Conflict of Interest

The authors declare that the research was conducted in the absence of any commercial or financial relationships that could be construed as a potential conflict of interest.
