# Supplementary material for: Identifying Drug Targets in Pancreatic Ductal Adenocarcinoma Through Machine Learning, Analyzing Biomolecular Networks, and Structural Modeling
Source: Front Pharmacol. 2020 Apr 30;11:534. doi: 10.3389/fphar.2020.00534 (PMC7204992; doi:10.3389/fphar.2020.00534)
Supplement: Supplementary file 1 [file DataSheet_1.docx]

Supplementary Material


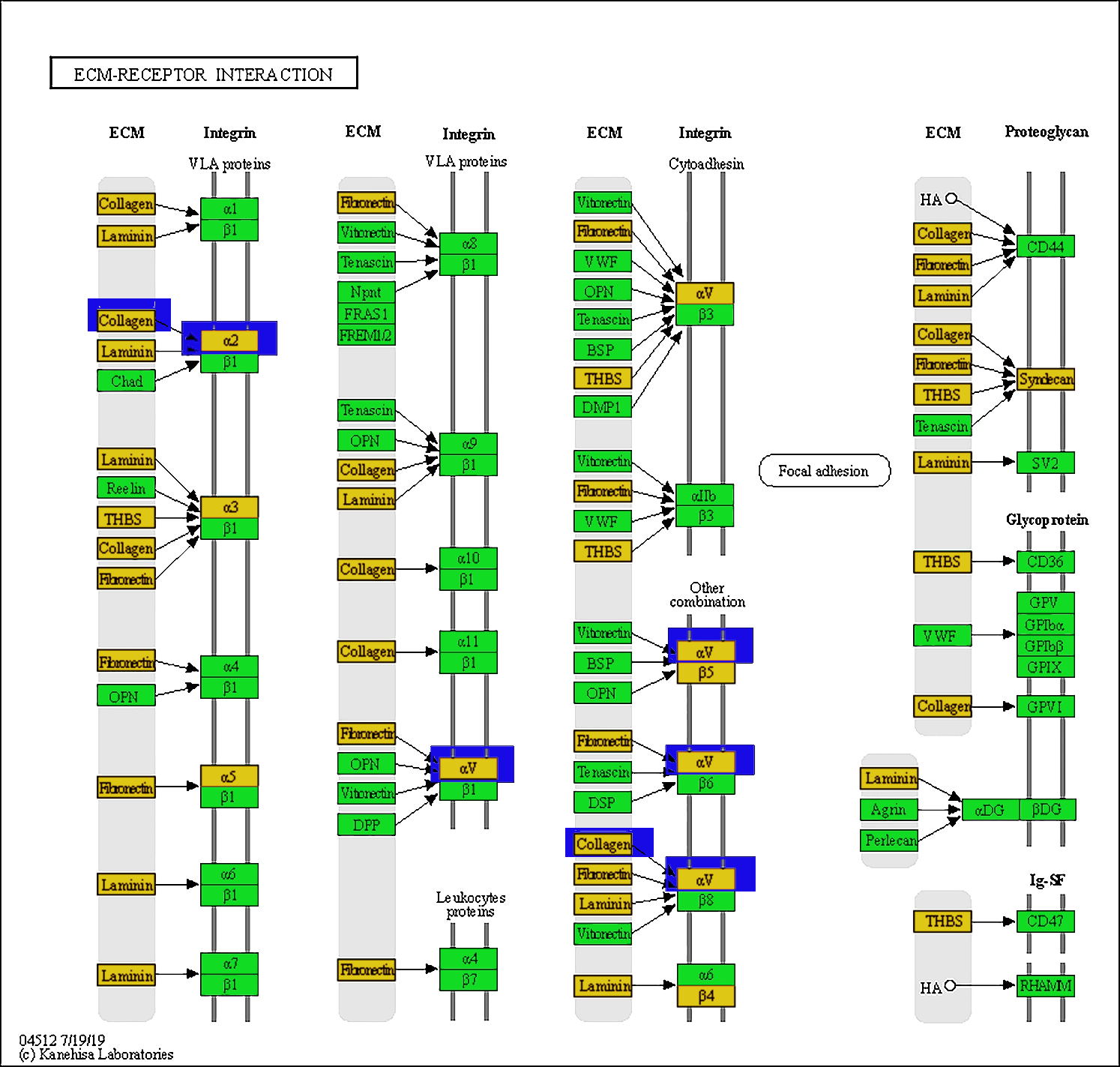


**Figure S1**. The ECM-receptor interaction pathway was retrieved from KEGG (https://www.genome.jp/dbget-bin/www_bget?map04512). Yellow boxes indicate PDAC DEGs, blue boxes indicate ITGAV, ITGA2, and their interactions with collagens.

**
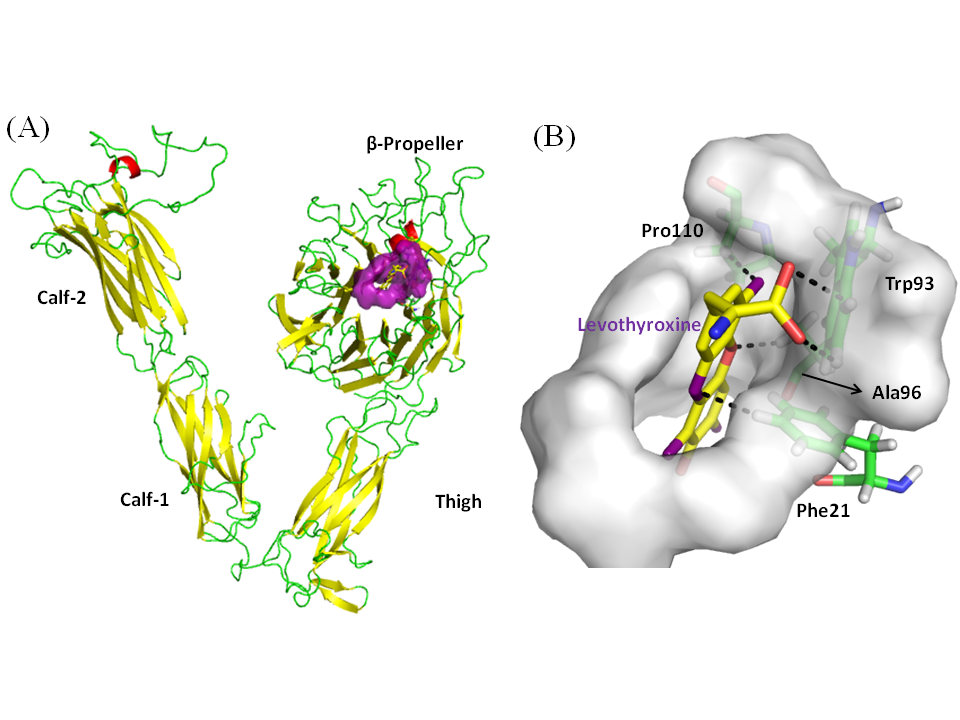
**

**Figure S2.** (A) The structure of ITGAV including the *β*-propeller, Thigh, Calf-1, and Calf-2 domains, and the second largest druggable pocket (purple), which is located at the at the hole of the *β*-barrel. (B) The binding poses by docking Levothyroxine into this pocket. Levothyroxine and the interacting residues are represented with colored sticks.

**Table S1.** The top 100 DEGs ranked by Rs value in each dataset.

| **GSE28735** | | | **GSE71989** | | | **GSE15471** | | |
| --- | --- | --- | --- | --- | --- | --- | --- | --- |
| **rank** | **ID** | **Rs** | **rank** | **ID** | **Rs** | **rank** | **ID** | **Rs** |
| 1 | AQP8 | 1.0000 | 1 | STX3 | 1.0000 | 1 | BTAF1 | 1.0000 |
| 2 | PLEK2 | 0.9992 | 2 | RAPGEF2 | 0.9997 | 2 | S100A14 | 0.9996 |
| 3 | DTL | 0.9984 | 3 | CD79B | 0.9994 | 3 | VCX2 | 0.9991 |
| 4 | RIC3 | 0.9975 | 4 | PPIC | 0.9990 | 4 | ACSM3 | 0.9987 |
| 5 | ABCC3 | 0.9967 | 5 | PMAIP1 | 0.9987 | 5 | CLIC4 | 0.9982 |
| 6 | ERN2 | 0.9959 | 6 | ARPC1B | 0.9984 | 6 | MKRN7P | 0.9978 |
| 7 | GPRC5A | 0.9951 | 7 | PCSK2 | 0.9981 | 7 | ARHGEF10 | 0.9973 |
| 8 | EPB41L5 | 0.9943 | 8 | OASL | 0.9977 | 8 | ODF3 | 0.9969 |
| 9 | WDHD1 | 0.9934 | 9 | OR6W1P | 0.9974 | 9 | OLFML2B | 0.9965 |
| 10 | FGF10 | 0.9926 | 10 | BST2 | 0.9971 | 10 | ROR2 | 0.9960 |
| 11 | CPB1 | 0.9918 | 11 | ZNF121 | 0.9968 | 11 | BAG2 | 0.9956 |
| 12 | ASB9 | 0.9910 | 12 | ZNF518A | 0.9964 | 12 | CLDN6 | 0.9951 |
| 13 | POSTN | 0.9902 | 13 | ARHGAP27 | 0.9961 | 13 | MARCKSL1 | 0.9947 |
| 14 | FABP4 | 0.9893 | 14 | CBR3 | 0.9958 | 14 | GNAL | 0.9942 |
| 15 | RRAS | 0.9885 | 15 | FTX | 0.9955 | 15 | BST2 | 0.9938 |
| 16 | HIST1H2BD | 0.9877 | 16 | SSR4 | 0.9951 | 16 | PROCR | 0.9934 |
| 17 | EDNRA | 0.9869 | 17 | BPGM | 0.9948 | 17 | DIP2B | 0.9929 |
| 18 | SFTA2 | 0.9861 | 18 | MGAT2 | 0.9945 | 18 | LARGE1 | 0.9925 |
| 19 | GATM | 0.9852 | 19 | S100A13 | 0.9942 | 19 | EPYC | 0.9920 |
| 20 | IFI44 | 0.9844 | 20 | LOC100129175 | 0.9938 | 20 | HIST1H2BD | 0.9916 |
| 21 | MLPH | 0.9836 | 21 | A2M-AS1 | 0.9935 | 21 | CLDND1 | 0.9911 |
| 22 | SULT1C2 | 0.9828 | 22 | SRRM5 | 0.9932 | 22 | IFIT1 | 0.9907 |
| 23 | DCBLD1 | 0.9820 | 23 | COPS7A | 0.9929 | 23 | KCNIP2 | 0.9903 |
| 24 | FAT1 | 0.9811 | 24 | LINC02003 | 0.9925 | 24 | MNDA | 0.9898 |
| 25 | LGALS1 | 0.9803 | 25 | ZBTB16 | 0.9922 | 25 | ZG16 | 0.9894 |
| 26 | SNORD116-14 | 0.9795 | 26 | CSK | 0.9919 | 26 | WDR1 | 0.9889 |
| 27 | PCDHB5 | 0.9787 | 27 | PEX1 | 0.9916 | 27 | MMP17 | 0.9885 |
| 28 | ADGRF1 | 0.9779 | 28 | TSPO | 0.9912 | 28 | IL10RA | 0.9880 |
| 29 | RASGRF2 | 0.9770 | 29 | ITGA4 | 0.9909 | 29 | MTMR11 | 0.9876 |
| 30 | IMPA2 | 0.9762 | 30 | DENND4B | 0.9906 | 30 | AATK | 0.9872 |
| 31 | PAH | 0.9754 | 31 | EML1 | 0.9903 | 31 | OLFM4 | 0.9867 |
| 32 | TMEM45B | 0.9746 | 32 | LOC100128993 | 0.9899 | 32 | TRHDE-AS1 | 0.9863 |
| 33 | SLC41A1 | 0.9738 | 33 | FARP2 | 0.9896 | 33 | PXDN | 0.9858 |
| 34 | BACE1 | 0.9729 | 34 | CD69 | 0.9893 | 34 | PPIA | 0.9854 |
| 35 | SERPINB9 | 0.9721 | 35 | CD37 | 0.9890 | 35 | ATP6V0C | 0.9849 |
| 36 | AGR2 | 0.9713 | 36 | SLCO6A1 | 0.9886 | 36 | MEST | 0.9845 |
| 37 | C1GALT1 | 0.9705 | 37 | PSG5 | 0.9883 | 37 | MT1X | 0.9841 |
| 38 | SLC30A8 | 0.9697 | 38 | DLGAP2-AS1 | 0.9880 | 38 | NT5C2 | 0.9836 |
| 39 | MTMR7 | 0.9688 | 39 | C19orf47 | 0.9877 | 39 | RASL11B | 0.9832 |
| 40 | LRRC15 | 0.9680 | 40 | RHOH | 0.9873 | 40 | DTX2P1-UPK3BP1-PMS2P11 | 0.9827 |
| 41 | MMRN1 | 0.9672 | 41 | TGM1 | 0.9870 | 41 | RBMS1 | 0.9823 |
| 42 | STEAP4 | 0.9664 | 42 | CA5B | 0.9867 | 42 | DSCAM | 0.9818 |
| 43 | ANO5 | 0.9656 | 43 | POLE4 | 0.9864 | 43 | ACTR2 | 0.9814 |
| 44 | NFIB | 0.9647 | 44 | CPSF4 | 0.9860 | 44 | SVIL | 0.9810 |
| 45 | HHEX | 0.9639 | 45 | MAP4K5 | 0.9857 | 45 | SPARC | 0.9805 |
| 46 | ELF3 | 0.9631 | 46 | CMTM7 | 0.9854 | 46 | PYGB | 0.9801 |
| 47 | PEX5L | 0.9623 | 47 | IRS2 | 0.9851 | 47 | ARPC2 | 0.9796 |
| 48 | RUNX1 | 0.9614 | 48 | PRTG | 0.9847 | 48 | S100A13 | 0.9792 |
| 49 | KIF14 | 0.9606 | 49 | IQCC | 0.9844 | 49 | CD160 | 0.9787 |
| 50 | FXYD3 | 0.9598 | 50 | TUBA1C | 0.9841 | 50 | SLC16A5 | 0.9783 |
| 51 | CACHD1 | 0.9590 | 51 | AP3B2 | 0.9838 | 51 | NEDD4 | 0.9779 |
| 52 | FAM3B | 0.9582 | 52 | VDR | 0.9834 | 52 | PKD2 | 0.9774 |
| 53 | ADAM22 | 0.9573 | 53 | APC | 0.9831 | 53 | CCN2 | 0.9770 |
| 54 | GLS2 | 0.9565 | 54 | HLA-DMB | 0.9828 | 54 | GREM1 | 0.9765 |
| 55 | APOBEC2 | 0.9557 | 55 | C22orf34 | 0.9825 | 55 | DUSP11 | 0.9761 |
| 56 | RHOF | 0.9549 | 56 | PDLIM3 | 0.9821 | 56 | GSK3A | 0.9756 |
| 57 | PMEPA1 | 0.9541 | 57 | SH3PXD2B | 0.9818 | 57 | LGALS1 | 0.9752 |
| 58 | ACSL5 | 0.9532 | 58 | STK39 | 0.9815 | 58 | CEACAM7 | 0.9748 |
| 59 | VCL | 0.9524 | 59 | KCNJ5 | 0.9812 | 59 | KLF5 | 0.9743 |
| 60 | TTLL7 | 0.9516 | 60 | SNX21 | 0.9808 | 60 | KCNK3 | 0.9739 |
| 61 | SDC1 | 0.9508 | 61 | TBCB | 0.9805 | 61 | ARL6IP1 | 0.9734 |
| 62 | TCEA3 | 0.9500 | 62 | SRPK2 | 0.9802 | 62 | USP18 | 0.9730 |
| 63 | PTPRR | 0.9491 | 63 | LY75 | 0.9799 | 63 | CDKN3 | 0.9725 |
| 64 | TRIM59 | 0.9483 | 64 | GNAT1 | 0.9795 | 64 | CRELD2 | 0.9721 |
| 65 | RTKN2 | 0.9475 | 65 | SEL1L | 0.9792 | 65 | AMHR2 | 0.9716 |
| 66 | GAS2 | 0.9467 | 66 | ALDH1L1-AS2 | 0.9789 | 66 | GNB5 | 0.9712 |
| 67 | CCN4 | 0.9459 | 67 | UBR5 | 0.9786 | 67 | RALGDS | 0.9708 |
| 68 | HEPACAM2 | 0.9450 | 68 | SUSD6 | 0.9782 | 68 | FAM216A | 0.9703 |
| 69 | GSTA1 | 0.9442 | 69 | LLGL1 | 0.9779 | 69 | DUSP7 | 0.9699 |
| 70 | CD80 | 0.9434 | 70 | ITGB3BP | 0.9776 | 70 | HLA-E | 0.9694 |
| 71 | HILPDA | 0.9426 | 71 | MTNR1B | 0.9773 | 71 | ZKSCAN5 | 0.9690 |
| 72 | GJC3 | 0.9418 | 72 | NR2E1 | 0.9769 | 72 | SERPINH1 | 0.9685 |
| 73 | PLAU | 0.9409 | 73 | NLE1 | 0.9766 | 73 | MYH14 | 0.9681 |
| 74 | SOX6 | 0.9401 | 74 | NAGA | 0.9763 | 74 | ITGA2 | 0.9677 |
| 75 | WDR17 | 0.9393 | 75 | MYBPC3 | 0.9760 | 75 | SHC1 | 0.9672 |
| 76 | AGMO | 0.9385 | 76 | AK1 | 0.9756 | 76 | CASP3 | 0.9668 |
| 77 | MMP3 | 0.9377 | 77 | TRIP11 | 0.9753 | 77 | CIB2 | 0.9663 |
| 78 | ITPR3 | 0.9368 | 78 | AATK | 0.9750 | 78 | RAB11A | 0.9659 |
| 79 | EGF | 0.9360 | 79 | CCNO | 0.9747 | 79 | KIAA0754 | 0.9654 |
| 80 | HOXB6 | 0.9352 | 80 | ITIH2 | 0.9743 | 80 | MICOS10P1 | 0.9650 |
| 81 | IRAK3 | 0.9344 | 81 | PYGB | 0.9740 | 81 | PSMB8-AS1 | 0.9646 |
| 82 | EPHA4 | 0.9336 | 82 | LOC105374690 | 0.9737 | 82 | CH25H | 0.9641 |
| 83 | LMO3 | 0.9327 | 83 | VPS9D1 | 0.9734 | 83 | OXR1 | 0.9637 |
| 84 | GNMT | 0.9319 | 84 | IL6 | 0.9730 | 84 | IER3 | 0.9632 |
| 85 | LGALS3 | 0.9311 | 85 | C1QB | 0.9727 | 85 | CPOX | 0.9628 |
| 86 | SLC2A2 | 0.9303 | 86 | MNT | 0.9724 | 86 | MCM3 | 0.9623 |
| 87 | KRT14 | 0.9295 | 87 | ZRSR2 | 0.9721 | 87 | ERO1A | 0.9619 |
| 88 | MYRIP | 0.9286 | 88 | SERPINI2 | 0.9717 | 88 | SLC25A45 | 0.9615 |
| 89 | SLC9A2 | 0.9278 | 89 | FOXM1 | 0.9714 | 89 | ALAS1 | 0.9610 |
| 90 | ITGA11 | 0.9270 | 90 | PDLIM1 | 0.9711 | 90 | RAB21 | 0.9606 |
| 91 | KLKB1 | 0.9262 | 91 | RFC3 | 0.9708 | 91 | ESRRG | 0.9601 |
| 92 | ADH1C | 0.9254 | 92 | TRPC1 | 0.9704 | 92 | VCAM1 | 0.9597 |
| 93 | CYP3A5 | 0.9245 | 93 | ACD | 0.9701 | 93 | EEF1AKMT3 | 0.9592 |
| 94 | ACADL | 0.9237 | 94 | MPDZ | 0.9698 | 94 | RBX1 | 0.9588 |
| 95 | SH3PXD2B | 0.9229 | 95 | EGF | 0.9695 | 95 | DNAJB6 | 0.9584 |
| 96 | ACTB | 0.9221 | 96 | LPIN2 | 0.9692 | 96 | SCGB2A1 | 0.9579 |
| 97 | ANXA2 | 0.9213 | 97 | CPSF6 | 0.9688 | 97 | BMPR2 | 0.9575 |
| 98 | PYGB | 0.9204 | 98 | PTPRO | 0.9685 | 98 | GPRC5A | 0.9570 |
| 99 | TSPAN1 | 0.9196 | 99 | FRRS1L | 0.9682 | 99 | CD209 | 0.9566 |
| 100 | CD82 | 0.9188 | 100 | DUOXA1 | 0.9679 | 100 | KCNJ5 | 0.9561 |

**Table S2.** Top 20 genes ranked by different parameters, including RNs, betweenness (BC_PPI), and closeness (CC_PPI) centralities.

| **RNs_GSE15471** | **RNs_GSE28735** | **RNs_GSE71989** | **B_PPI** | **C_PPI** |
| --- | --- | --- | --- | --- |
| ADAM10*** | ADAM10*** | TIMP1*** | EGF** | FN1* |
| ITGA2** | EGF** | EGF** | FN1* | EGF** |
| LGALS1** | SDC1* | ITGAV** | ADAM10*** | ADAM10*** |
| TIMP1*** | FN1* | ALB** | ITGAV** | TIMP1*** |
| MATN3*** | LGALS1** | MFGE8** | EDNRA | ITGAV** |
| PKM*** | APOL1** | CALU*** | STAT1 | ALB** |
| APLP2*** | APLP2*** | ADAM10*** | SFN | ACTN1*** |
| COL5A1* | TIMP1*** | MATN3*** | LAMC2 | SDC1* |
| ACTN1*** | PLAU* | ITGA2** | MMP9 | ITGA2** |
| CALU*** | PLAUR* | VCAN*** | CDK1 | SDC4 |
| CTNNA1* | IGFBP3* | MSLN** | SDC1* | CTNNA1* |
| VCAN*** | VCAN*** | APOL1** | LYZ | VCAN*** |
| BST2** | ALB** | COL3A1* | ITGB4 | PLAUR* |
| MX1* | FBN1* | APLP2*** | ACTN1*** | LAMC2 |
| COL1A2* | PKM*** | PKM*** | JUP | PLAU* |
| COL6A3* | ACTN1*** | BST2** | TIMP1*** | ITGB4 |
| MFGE8** | CALU*** | ITGB5* | CEACAM6 | FBN1* |
| SPARC* | COL1A1* | ACTN1*** | FBN1* | MMP9 |
| ITGAV** | MSLN** | ITGA5* | ITGA2** | MFGE8** |
| IFI27* | MATN3*** | MX2* | CTNNA1* | ACTN4 |

***in all three datasets

**in two of the three datasets

*in one of the three datasets
